# Patterned Illumination Techniques in Optogenetics: An Insight Into Decelerating Murine Hearts

**DOI:** 10.3389/fphys.2021.750535

**Published:** 2022-01-11

**Authors:** Laura Diaz-Maue, Janna Steinebach, Claudia Richter

**Affiliations:** ^1^Department of Research Electronics, Max-Planck-Institute for Dynamics and Self-Organization, Göttingen, Germany; ^2^Biomedical Physics Research Group, Max-Planck-Institute for Dynamics and Self-Organization, Göttingen, Germany; ^3^German Center for Cardiovascular Research (DZHK e., V.), Göttingen, Germany; ^4^Laboratory Animal Science Unit, German Primate Center, Leibniz-Institute for Primate Research, Göttingen, Germany

**Keywords:** cardiac optogenetics, channelrhodopsin-2, arrhythmia, photostimulation, feedback techniques, arrhythmia classification, deceleration

## Abstract

Much has been reported about optogenetic based cardiac arrhythmia treatment and the corresponding characterization of photostimulation parameters, but still, our capacity to interact with the underlying spatiotemporal excitation patterns relies mainly on electrical and/or pharmacological approaches. However, these well-established treatments have always been an object of somehow heated discussions. Though being acutely life-saving, they often come with potential side-effects leading to a decreased functionality of the complex cardiac system. Recent optogenetic studies showed the feasibility of the usage of photostimulation as a defibrillation method with comparatively high success rates. Although, these studies mainly concentrated on the description as well as on the comparison of single photodefibrillation approaches, such as locally focused light application and global illumination, less effort was spent on the description of excitation patterns during actual photostimulation. In this study, the authors implemented a multi-site photodefibrillation technique in combination with Multi-Lead electrocardiograms (ECGs). The technical connection of real-time heart rhythm measurements and the arrhythmia counteracting light control provides a further step toward automated arrhythmia classification, which can lead to adaptive photodefibrillation methods. In order to show the power effectiveness of the new approach, transgenic murine hearts expressing channelrhodopsin-2 *ex vivo* were investigated using circumferential micro-LED and ECG arrays. Thus, combining the best of two methods by giving the possibility to illuminate either locally or globally with differing pulse parameters. The optical technique presented here addresses a number of challenges of technical cardiac optogenetics and is discussed in the context of arrhythmic development during photostimulation.

## 1. Introduction

Ventricular arrhythmias are not only complex in clinical practice but also pose a challenge in the exploration and optimization of new, gentler termination approaches. In particular, the bar is set high for exploratory, experimental studies in characterizing the underlying modes of function and the resulting translational approaches.

It seems that the best known clinical approach is the global termination of ventricular tachyarrhythmia by delivery of high-energy electrical shocks, either externally or internally, which have been shown to be very effective and, most importantly, fast-acting. However, especially in cases of frequently necessary or incorrect application, considerable side effects, such as electroporation and traumatic tissue damages (Moss et al., 1996; Tokano et al., 1998), make it necessary to optimize this method. In the course of this, termination protocols with more than one shock or more specific electrical pulses have come into focus. The application of multiple less energetic pulses can counteract the arrhythmia in its origin as a circularly propagating cardiac excitation (Efimov et al., 2000; Exner, 2005), as e.g., already applied as anti-tachycardia pacing (ATP) in patients (see e.g., Wathen et al., 2004). Furthermore, there are also experimental, pre-clinical multi-pulse protocols since such protocols have a significant influence on synchronization of excitation patterns (Exner, 2005), which try to reach the minimum necessary energy for a successful termination in different approaches, on the one hand with changing pacing frequency or on the other hand with changing amplitude level (Pumir et al., 2007; Luther et al., 2011; Janardhan et al., 2012).

Though, the majority of experimental studies face the hurdle that during the administration of electrical pulses, the measurement systems, such as the electrocardiogram (ECG), get “blinded,” due to the artifacts caused by either the defibrillation or pacing pulses applied. To circumvent this, optical mapping is frequently used, which itself sometimes exerts strong influences on the cardiac system, e.g., through the use of chemicals such as fluorescent dyes or corresponding mechanical uncouplers (see e.g., Kolega, 2004; Swift et al., 2012; Zaglia et al., 2015). In this context, cardiac optogenetics and photostimulation exhibit great advantages. Its modus operandi is not based on electrical pulses, but on light pulses, so that there are no negative influences on the ECG to be expected. When using transgenic animal models, the genetic expression distribution of the optogenetic sensors/channels is homogeneous or locally specified. In addition, the application of non-electrical pulses is also less susceptible to short-term side effects such as electroporation. Lastly, since the light pulses can be delivered in a locally specific manner, cardiac regional success differences in termination can be further investigated with globally acting protocols.

In this study, the authors intend to show that cardiac optogenetics is a valid experimental tool for the investigation of arrhythmia behavior during pacing, which can provide information about the termination probability of the arrhythmia. To characterize and visualize this, a new multi-ECG setup was developed, making possible to observe different multi-pulse protocols with respect to their termination success and effects on the arrhythmia. Finally, the possibility of such a system for heart-specific arrhythmia termination will be discussed.

## 2. Materials and Methods

Experiments involving lab animals were performed in accordance with the current version of the German animal welfare law and reported to our animal welfare representatives. Application for approval has been approved by the responsible animal welfare authority (Lower Saxony State Office for Consumer Protection and Food Safety). Moreover, humane welfare-oriented procedures were carried out in accordance with the Guide for the Care and Use of Laboratory Animals and were performed after recommendations of the Federation of Laboratory Animal Science Associations (FELASA).

### 2.1. Langendorff Perfusion

This study was accomplished using a constitutive transgenic mouse model, α-MHC-ChR2, at the age of 17 weeks and older, whereby ChR2 expression was proven by biomolecular protocols and restricted exclusively to cardiac tissue. In the experiment, a retrograde *ex vivo* perfusion after Langendorff was used. For this method, as described elsewhere (Richter et al., 2016), the murine heart was explanted, afterwards cannulated *via* the aorta and perfused with Tyrode's solution (130 mM NaCl, 4 mM KCl, 1 mM MgCl_2_, 24 mM NaHCO_3_, 1.8 mM CaCl_2_, 1.2 mM KH2PO_4_, 5.6 mM glucose, and 1% albumin/BSA; aerated with carbogen (5% CO_2_ and 95% O_2_)). A constant flow rate of (2.63 ± 0.58) ml/min was applied, whereby normal heart rate varied from 4 to 6 Hz and arrhythmic frequencies were observed between 15 and 30 Hz.

For experimental induction of arrhythmic patterns, normal perfusion was switched to low-K+ Tyrode's solution (130 mM NaCl, 2 mM KCl, 1 mM MgCl2, 24 mM NaHCO3, 1.8 mM CaCl2, 1.2 mM KH2PO4, 5.6 mM glucose, and 1 % albumin/BSA were aerated with carbogen (95 % oxygen and 5 % CO 2)) with 100 μM Pinacidil as described by Bruegmann et al. (2016). In contrast to other publications, Richter et al. (2016), Quiñonez Uribe et al. (2018), and Sasse et al. (2019) the initiation of arrhythmia in this work was induced with rapid optical pacing as also shown in Diaz-Maue et al. (2021). Sustained arrhythmias were achieved when remaining for *t*_*term*_ = 5 s, control experiments which confirm this criterion are further detailed in section 3.2. Should the induced arrhythmia terminate within *t*_*term*_, it was classified as self-terminated. In addition, if an arrhythmia failed to be terminated at the fifth photostimulation attempt, then backup was used. The backup consisted of activating all μLED arrays at the same time at the highest experimentally used light intensity resulting in global illumination.

### 2.2. Multi-Lead ECG and μLED Arrays

**Figure 1:**
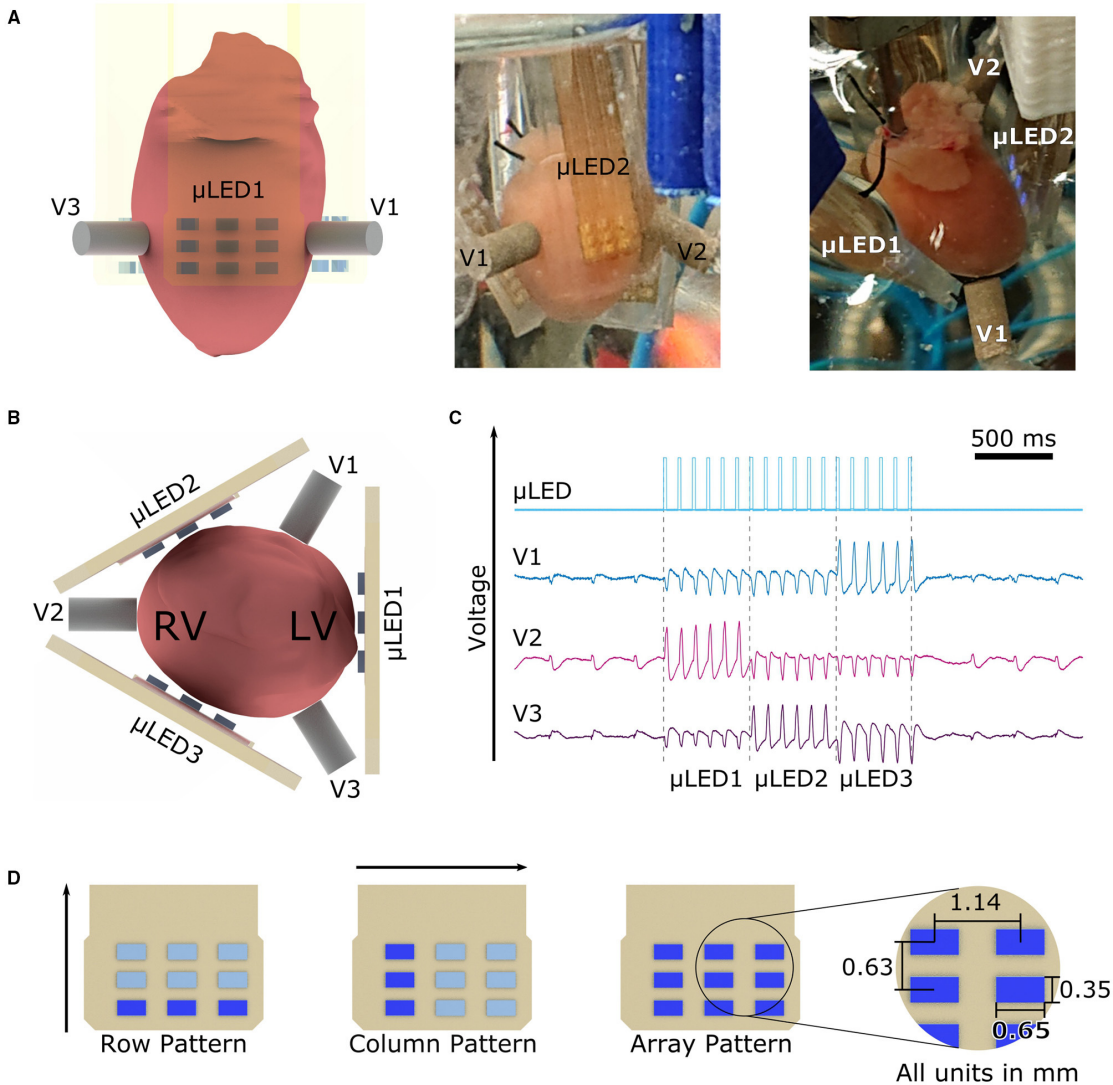
Experimental setup with three lead ECGs, three μLED arrays, and illumination patterns used. **(A)** The murine heart was encompassed by three μLED arrays and three ECG Electrodes as shown in all panels from different angles. **(B)** μLED1 was always positioned onto the left ventricle of the heart, following μLED arrays were positioned with a 120° angle with respect to each other. In a similar way, the ECG electrodes V1, V2, and V3 are positioned in the space between μLED arrays. **(C)** ECG recording showing the photostimulation given by the μLED array and the corresponding responses measured by V1, V2, and V3 electrodes. The change of polarity, as well as the change in amplitude, demonstrate that the chosen measuring electrode registers the highest potential when the opposite μLED array is activated **(D)** Applied illumination patterns. The row pattern initiates illumination in the lower row of every array at the given frequency and pulse width, the column pattern starts with the left column and rotates around the heart with the parameters set. The array pattern activated the nine μLEDs at the same time in μLED1 then turned off after the given pulse width, similarly, μLED2 was then activated, and finally μLED3 irradiated the heart at last. The spacing of the μLED in x-direction is 1.14 mm and in y-direction is 0.63 mm.

A custom-built Multi-Lead ECG device was designed to simultaneously record cardiac activity from different regions of the heart. Based on the Wilson Hypothesis by Wilson et al. (1934, 1946), a Wilson Central Terminal (WCT) was built by connecting three surface Ag/AgCl electrodes V1, V2, and V3 to a common point. The potential difference between these three electrodes and the WCT are referred to in this work as uni-polar lead measurements. During the experiments, the three electrodes were positioned at an angle of 120° as shown in Figures 1A,B. The signals acquired were digitally converted and afterward analyzed.

Motivated by the work of Schwaerzle et al. (2014) and Diaz-Maue et al. (2018, 2021), a set of custom-built micro-LED (μLED) arrays were designed. Briefly, blue LEDs (XZBBR155W5MAV, SunLED, USA) with a central wavelength λ_*blue*_ = 470 nm were reflow soldered onto a printed circuit board (PCB). The material used for the PCB was a prepreg laminate (104 ML, LPKF, Garbsen, Germany) with a thickness of 200 μm. The blue LEDs were arranged in a 3 × 3 array with a horizontal distance of 1.14 mm as shown in Figure 1D, yielding to an active illumination area of 4.76 mm^2^. The conductive tracks on the PCB were isolated embedding the complete array into a layer of Polydimethylsiloxane (PDMS, Sylgard 184, Dow Corning, USA). PDMS was chosen because of its excellent biocompatibility, long-term stability, and electrical isolation properties (Hamelink, 1992). The optical characterization of the μLED arrays was performed with a PM100D optical power meter (Thorlabs, Germany) and the S120VC photodiode power sensor (Thorlabs, Germany). In this study, three μLED arrays (μLED1, μLED2, and μLED3) were positioned as well as the ECG electrodes in a 120° angle encircling the heart (cf. Figure 1B). The current flowing through the resulting 27 μLEDs was controlled *via* the lab computer using a custom-made μLED-Driver. Control and analysis were realized with a custom-software written in Python (Python Software Foundation, USA).

The proof-of-working of the system was achieved by conducting an experiment measuring pacing thresholds (refer to Supplementary Figure 1). As it can be seen in Figure 1C, no electrical artifacts were induced in any uni-polar lead during photostimulation. This confirms that the PDMS layer provided adequate isolation for the electrical tracks on the PCB. Figure 1C shows as well, that the pacing signal of every μLED array is measured in the opposite electrode, i.e., when μLED1 illuminates the heart, the electrical activity is recorded in V2. In this scenario, since all three electrodes are forming the WCT terminal (zero voltage point), the change of potential that is caused by the expanding electrical wave initiated by μLED1 is measured by V1 and V3, these two contribute to the WCT equally due to their proximity, as a consequence, the maximum change of potential is then recorded by electrode V2.

### 2.3. Illumination Patterns

Inspired by the theory of excitable media, three different illumination patterns, namely row, column, and array protocols, were designed to explore the most effective way of breaking or blocking the arrhythmia-generating spiral waves. The motivation for the row protocol was to drive the spiral wave(s) upward toward the atria, so that the uncoordinated electrical activity collides with non-excitable tissue, getting thereby terminated. As for the column and array protocol, it was intended to evoke a conduction block to terminate the arrhythmia by first exciting a defined area of tissue, consequently leading to a refractory period and therefore causing the spiral wave to be absorbed or blocked at the boundary. The column protocol illuminates only a small area of the heart at once, thus putting several successive segments into the non-excitable state. On the contrary, the array protocol illuminates larger areas at once thereby limiting the number of non-excitable segments to three. In this study, these three approaches were compared to determine which is more effective in terminating arrhythmia.

Accordingly, these three experimental protocols were applied in order to determine illumination patterns that optimize defibrillation success in terms of defibrillation energy and frequency of illumination. Furthermore, it was investigated whether the applied protocols were especially suitable for certain arrhythmia types or arrhythmia frequencies.

In the row protocol, the bottom three μLEDs of all three arrays were activated simultaneously, so that the illumination formed a ring around the heart. Afterwards, the two rows above followed one after the other with one pulse each. In this study, five repetitions of the mentioned sequence were used to deliver a total of *k* = 15 pulses per attempt. During the column protocol, first the very left column of μLED1 emitted one pulse. Subsequently, the middle and right column of μLED1 followed, afterwards μLED2 and μLED3 continued with the same pattern, respectively. In this case, the protocol was repeated only two times, consequently the light pulses circulated two times around the surface of the heart in a counterclockwise direction resulting in *k* = 18 pulses. The last protocol tested was the array protocol. Hereby, the entire μLED1 was activated to deliver five pulses to the heart. μLED2 and μLED3 followed, emitting five pulses each as well. In this way, the heart was circled only one time with a total number of *k* = 15 pulses. All illumination patterns are illustrated in Figure 1D.

In order to investigate the effect of light intensity, three different radiant fluxes, Φ = (1.7 ± 0.1) mW, (2.4 ± 0.1) mW and (3.3 ± 0.1) mW, were chosen for all three illumination patterns. Furthermore, five different pulse frequencies *f*_*stim*_ = 18, 20, 22, 24, and 26 Hz were tested to assess whether the chosen stimulation frequency *f*_*stim*_ influences the success of termination. The pulse duration of each pulse was kept constant at *d*_*stim*_ = 20 ms since this value proved to be particularly successful in earlier experiments (Diaz-Maue et al., 2021). The energy applied for every arrhythmia termination attempt *Q* was calculated as the product of the number of pulses *k*, the radiant flux Φ, and the pulse duration *d*_*stim*_, yielding values between *Q*_*min*_ = (0.51 ± 0.03) mJ and *Q*_*max*_ = (1.19 ± 0.03) mJ (cf. Supplementary Figure 2).

Finally, a positive control group was introduced with *N*_*m*_ = 4 mice. Defibrillation attempts were performed with global illumination using three high-power LEDs arranged as described in Quiñonez Uribe et al. (2018). Here, the light intensity was *LI*= 1 mW/mm^2^ which corresponds to a radiant flux of Φ = (58 ± 5) mW. Similarly to the three proposed protocols, photostimulation was performed with *k* = 15 pulses, pulse width *d*_*stim*_ = 20 ms, and frequencies of *f*_*stim*_ = 18, 20, 22, 24, and 26 Hz. This resulted in an energy of photostimulation of *Q* = (52.2 ± 4.5) mJ.

### 2.4. Arrhythmia Classification

In cardiac tissue, ventricular tachyarrhythmia is subdivided into tachycardia (VT) and fibrillation (VF). In order to correctly assess the successes or failures of photodefibrillation attempts, it is necessary to classify the individual arrhythmia into different categories depending on their complexity.

The obtained ECG recordings were post-processed after the experimental series to classify the arrhythmia type. For this purpose, a segment of every uni-polar lead measurement was extracted 1 s before every photostimulation attempt. Three different procedures were applied to determine the complexity of an arrhythmia. First, the evaluation of the morphology of the ECG signal according to the updated Lambeth convention (Curtis et al., 2013) was considered. Second, a sine fit was applied to the ECG recordings to identify the dominant frequency of the arrhythmia. The sine fit was implemented in the mathematical language Python by means of a curve fit that uses non-linear least squares to fit a sine function to the data. Amplitude, frequency, and phase were thereby fitted simultaneously. The sine fit represents an automated version of the periodicity analysis, as similarly performed by Skanes et al. (1998) in Langendorff perfused sheep hearts. Third, the Fast Fourier Transform (FFT) in combination with a Lorentzian fit was employed. The resulting spectrum of the FFT was used to estimate the dominant frequency of the arrhythmia as well as to investigate the variety of frequencies involved, as also described by Skanes et al. (1998). Based on the results of these methods, all arrhythmia were classified into three categories, namely monomorphic ventricular tachycardia (mVT), polymorphic ventricular tachycardia (pVT), and ventricular fibrillation (VF), with spatiotemporal complexity increasing from mVT to pVT and VF (Curtis et al., 2013).

**Figure 2:**
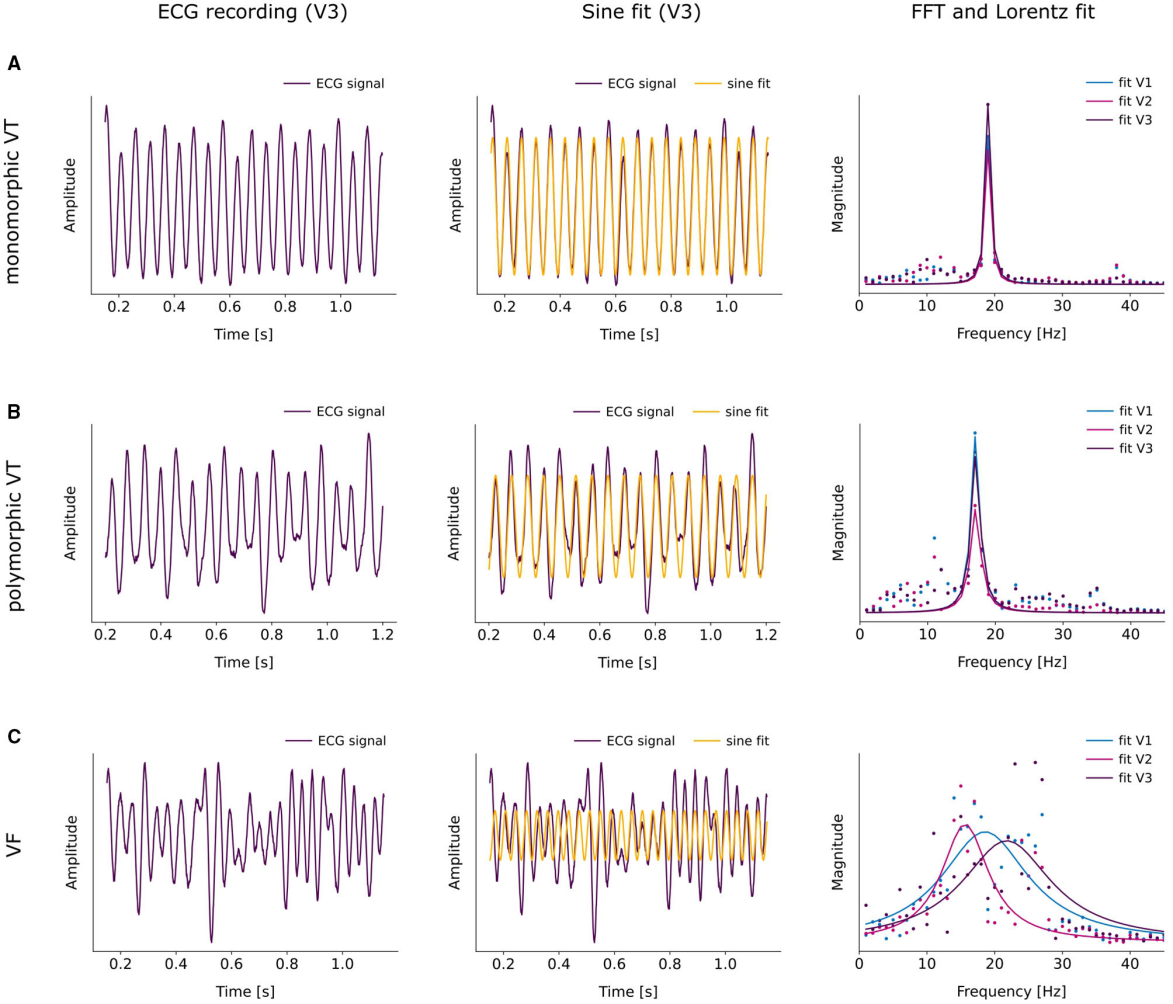
Representative examples of the classification of the three arrhythmia types mVT **(A)**, pVT **(B)** and VF **(C)**. The left column shows the ECG recording acquired by electrode V3, the middle column displays the applied sine fit to the corresponding ECG signal and the right column illustrates the fitting of a Lorentzian function to the FFT of the ECG signal. **(A)** Example of a monomorphic VT. The sine fit results in a dominant frequency of *f*_*dom*_ = (19.2 ± 0.5) (*gof* = 0.8). The FFTs and Lorentzian fits of the three ECG signals of V1, V2 and V3 agree to a large extent. They yield the dominant frequencies *f*_*dom,V*1_ = (19.0 ± 0.1) Hz and *fwhm* = (0.9 ± 0.1). **(B)** Example of a polymorphic VT. According to the sine fit, the dominant frequency of the arrhythmia lies at *f*_*dom*_ = (17.3 ± 0.4) Hz with *gof* = 0.6. The Lorentzian fits to the FFT spectra result in *f*_*dom*_ = (17.1 ± 0.1) Hz for all three ECG signals with slightly varying widths *fwhm*_*V*1_ = (1.5 ± 0.2), *fwhm*_*V*2_ = (1.8 ± 0.3) and *fwhm*_*V*3_ = (1.6 ± 0.3). **(C)** Example of a VF. Sine fitting the ECG signal of V3 gives a dominant frequency of *f*_*dom*_ = (26.4 ± 0.3) Hz at *gof* = 0.1. The Lorentzian fit to the FFT of the the ECG signals of V1, V2 and V3 provides rather distinct peaks with variable widths, resulting in *f*_*dom,V*1_ = (18.6 ± 0.7) Hz, *f*_*dom,V*2_ = (15.6 ± 0.5) Hz, *f*_*dom*_ = (21.8 ± 1.1) Hz and *fwhm*_*V*1_ = (15.7 ± 2.2), *fwhm*_*V*2_ = (8.4 ± 1.4), *fwhm*_*V*3_ = (16.2 ± 3.2).

Figure 2 shows illustrative examples of the three types of arrhythmia. The ECG representative recordings shown here were all acquired by electrode V3. Clearly, it is shown that the ECG turns into a more irregular pattern the more complex the arrhythmia becomes.

Accordingly, the sine fit for the rather regular mVT is almost in perfect agreement with the ECG signal, apart from slight variations in the amplitude (cf. Figure 2A). Furthermore, the increasing complexity of the arrhythmia leads to the occurrence of moderate deviations of the fit to the ECG signal in pVT (cf. Figure 2B) and to major deviations in VF (cf. Figure 2C). In order to quantify the observed deviations, the sine fit algorithm computes the normalized difference between the fitted signal and the original signal, in addition to the optimal parameters for amplitude and frequency. This value is then subtracted from 1 and the self-defined indicator “goodness of fit” (*gof*) is obtained. When *gof* is close to 1, the fit is more precise. Thereupon, the following intervals were defined, *gof* > 0.6 for mVT, 0.2 ≤ *gof* ≤ 0.6 for pVT, and finally, *gof* < 0.2 for VF.

Moreover, an FFT was performed to evaluate the ECG signal in the frequency domain. Since the FFT returns a spectrum of frequencies that can be detected in the original signal, a Lorentzian fit was applied to quantify the relevance of the different frequencies. The values for the center of the fit and the full width half max (*fwhm*) gave information about the dominant frequency and the width of the fit indicating if there was only one dominant frequency or if several frequencies contributed substantially. Accordingly, if 1 < *fwhm* < 10, the arrhythmia was classified as pVT, as mVT if *fwhm* ≤ 1, and as VF if *fwhm* ≥ 10.

In the case of the mVT in Figure 2A, the Lorentzian curves fitted to the FFT of the ECG signals of V1, V2, and V3 were very narrow and coincide for all three ECG signals. Figure 2B corresponds to a pVT, in this example, the Lorentzian curves were broader and slightly different for each electrode. Furthermore, components of different frequencies could be observed alongside the main peak. Notably, the Lorentzian fits varied substantially for the VF shown in Figure 2C. It is therefore clear that the three electrodes V1, V2, and V3 recorded ECG signals that contain distinct frequency information, which is reflected in the large width of the curves.

The described analysis was performed for all three uni-polar lead recordings. Should one of the three mentioned methods suggest different arrhythmia types, the whole arrhythmia was classified according to the most complex type.

### 2.5. Time-Frequency Analysis

Obtaining information about the frequency content of a signal is a usual task of the FFT (Chorro et al., 2006; Nash et al., 2006; Masse et al., 2007; Umapathy et al., 2010; Caldwell et al., 2012) or of the Short-Time Fourier Transform STFT (Tseng and Tseng, 2020; Coult et al., 2021). However, both require stationary signals (Clayton and Murray, 1993; Mansier et al., 1996; Seely and Macklem, 2004) and are therefore only suited for providing information about a signal as a whole. In the case of cardiac arrhythmia, the obtained signals contain short-term transients which cannot be completely described in the frequency domain alone and also require temporal resolution. Accordingly, to do a comprehensive evaluation of the transitory events in the ECG recordings, especially during photodefibrillation, the continuous wavelet transform (CWT) was chosen to deliver information in the combined time-frequency domain as also described by Torrence and Compo (1998), Abbate et al. (2002). The CWT signal processing was done using MATLAB (Mathworks, USA). First, all photodefibrillation attempts were localized and the recorded signal was shortened to 3 s before and 3 s after photostimulation, where the points *t*_*o*_ and *t*_*f*_ were defined, respectively, as the time for the first and last light pulse given. Afterwards, the CWT was calculated using a Morse wavelet as mother wavelet and the corresponding magnitude scalograms were plotted. Second, in order to obtain the frequencies which correspond to the largest scales present in the CWT, the maximum scale *a*_*max*_(*t*_*s*_(*i*)) for every sampling point *t*_*s*_(*i*) was computed, then all scales in the scalogram matrix *a* which were larger than 0.8·*a*_*max*_(*t*_*s*_(*i*)) were extracted. Following this criteria, the frequencies corresponding to *a* > 0.8·*a*_*max*_ were obtained yielding an array with several values for every *t*_*s*_(*i*). The mean value of every array was then calculated yielding to $\bar{fc}\left(t_{s}\left(i\right)\right)$ which represents now the instantaneous frequency for every point at time *t*_*s*_. Furthermore, the dominant frequencies for every time range of interest, namely, before *fc*_*bef*_, during *fc*_*dur*_, and after *fc*_*aft*_ photodefibrillation were calculated as the mean value of $\bar{fc}\left(t_{s}\left(i\right)\right)$ during the required intervals, defined as *before* *t*_0_ − 3 ≤ *t*_*s*_*bef*__ ≤ *t*_0_, *during* *t*_0_ < *t*_*s*_*dur*__ ≤ *t*_*f*_, and *after* *t*_*f*_ < *t*_*s*_*aft*__ ≤ *t*_*f*_ + 3. The same method was applied for every ECG recording acquired, generating the values *fc*_*bef*(1−3)_, *fc*_*dur*(1−3)_, and *fc*_*aft*(1−3)_.

## 3. Results

### 3.1. Arrhythmia Distribution and Characteristics

Performing the arrhythmia classification as mentioned in section 2.4 for the total number of recorded arrhythmia *N*_*arr*_ = 458 from *N*_*m*_ = 11 mice, it was found that the majority of events, namely 55 % were classified as pVTs. mVT and VF events made up 24% and 21%, respectively. Regarding the three illumination patterns, ${\overset{-}{N}}_{arr}=143\pm 16$ arrhythmias were acquired per pattern.

In order to define successful termination, it was examined whether an arrhythmia terminated simultaneously with the end of illumination or whether it continued beyond the point *t*_*f*_. In case of the latter, the time until the ECG signal returned to the base line was denoted as transient time. About half of all recorded arrhythmia exhibited transient times ranging between 50 and 2200 ms. Since 90 % of all arrhythmia with transients were terminated within 1300 ms after the end of illumination, it was defined that transient times which were less than or equal to 1300 ms correspond to successful termination whereas transient times larger than 1300 ms were classified as non-successful.

### 3.2. Termination Success

**Figure 3:**
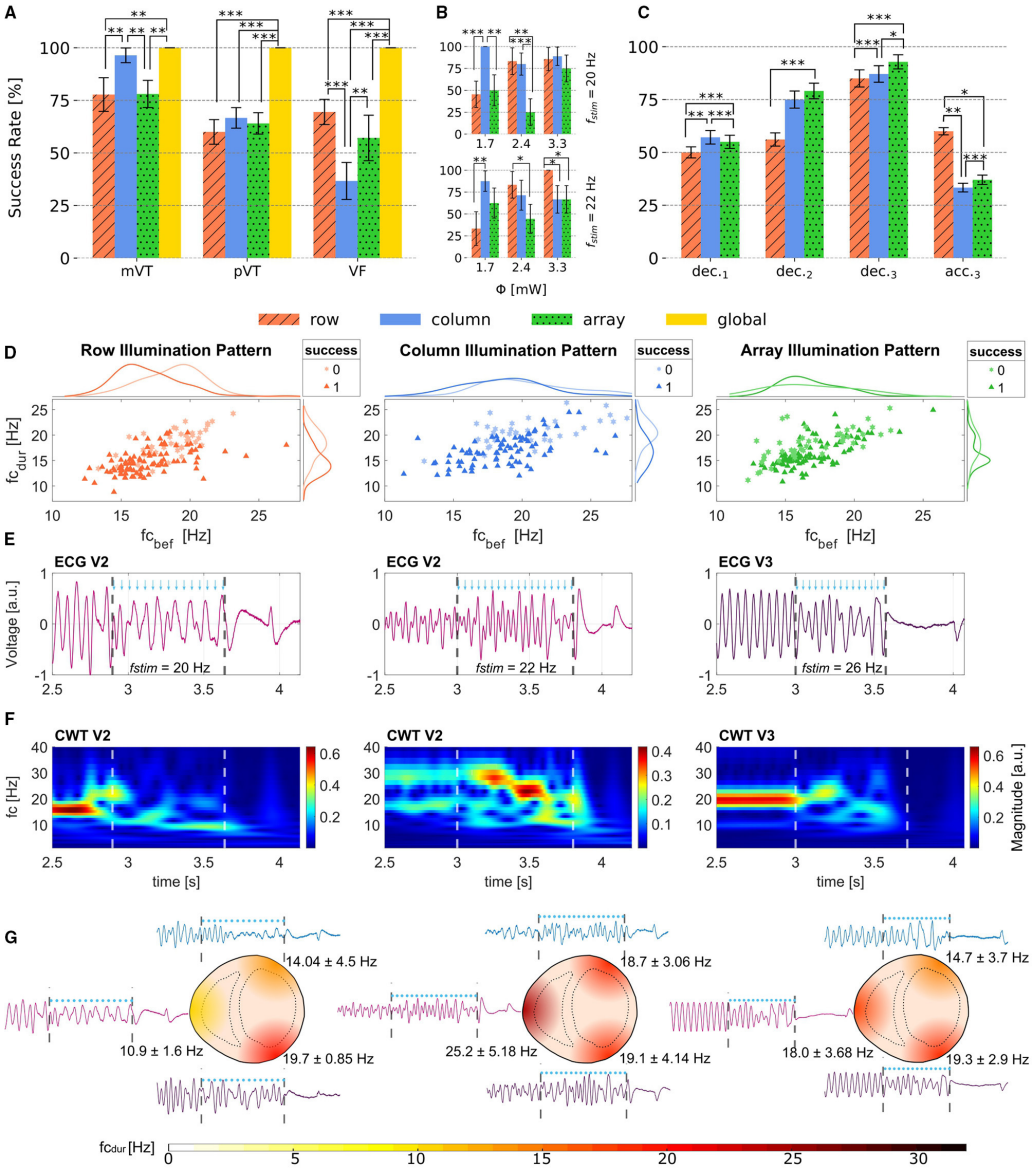
Obtained Success Rates and effects of illumination during arrhythmia. **(A)** Success rates for mVT, pVT and VF depending on the illumination pattern. **(B)** Success rates for the photostimulation frequencies *f*_*stim*_ = 20 and 22 Hz and the three different radiant fluxes applied. While the array protocol has the lowest termination rate for this two selected *f*_*stim*_, the row pattern terminates better with *f*_*stim*_ = 20 Hz and the lowest radiant flux applied, and the column pattern reaches a success rate of 100% when applying 3.3 mW radiant flux and *f*_*stim*_ = 22 Hz. **(C)** Termination rates shown with the deceleration observed in 1,2 and 3 Leads or acceleration measured in 3 leads. The data shown in **(A–C)** is reported with Error bars calculated with Standard Error of Mean (SEM). **(D)** Scatter plots showing the relation between *fc*_*bef*_ and *fc*_*dur*_ for row, column and array patterns. In addition the marginal histograms show the occurrence of success and failed attempts. **(E)** ECG recordings from different electrodes for one successful event using the three illumination patterns. The blue arrows show the timing of the light pulses and the dashed lines show the interval during illumination. **(F)** Magnitude Scalograms from the ECG leads shown in **(E)**. It can be observed that all three protocols are able to change both frequency and amplitude of the arrhythmia. **(G)** Representation of the heart for row (left), column (center) and array (right) protocols illustrating the extracted frequencies *fc*_*dur*_ from V1, V2, and V3. Statistical significance illustrated as *p < 0.1, **p < 0.05 and ***p < 0.01.

When considering the success rates of photodefibrillation regarding the three different illumination patterns without further distinguishing the parameters frequency of stimulation *f*_*stim*_ or applied radiant flux Φ, it became apparent that for mVTs, the column protocol worked most efficiently, since it was able to terminate (96.4 ± 1.7) % of all mVT independently of the selected parameters (see Figure 3A). The row and array protocol both showed a lower success rate around 78 % for mVT. In comparison, no such clear trend emerged for the pVT events. All protocols showed similar success rates for this arrhythmia type, on average (63.6 ± 4.5) %. Moreover, the column illumination pattern offered the lowest success rate of (37 ± 9) % for VF events. Regarding the row protocol, it is remarkable that it terminated VF events more reliably than pVT even though VF represent the more complex arrhythmia type.

In summary, these results indicate that different illumination patterns could be suited for different arrhythmia types. Figure 3A shows that the row protocol is overall most efficient for VF, whereas the column protocol should be chosen when attempting to terminate mVT. During pVT, all illumination patterns worked comparably well. In contrast, the control group employing global illumination terminated 100 % of *N*_*arr*_ = 75 independently of their type. In order to confirm the significance of the presented results, statistical tests were made using a one-tailed *z*-test for proportions as shown in Figure 3A.

Unfortunately, the sample size for VF events proved to be too limited to provide meaningful results once a further distinction according to the stimulation parameters was to be made. This is a consequence of the circumstance that it was not possible to determine the arrhythmia type during the experiments, leading to a randomized distribution of mVT, pVT, and VF. For further evaluation, mVT and pVT were combined into the group of VT, because this supports a sufficient sample size of arrhythmia when analyzing the success of termination based on different parameters. Considering the individual illumination patterns, the mean number of VT for evaluation per protocol type lies at ${\overset{-}{N}}_{VT}=116\pm 14$.

In order to analyze the dependence on the applied radiant flux Φ, the termination success was investigated for two fixed stimulation frequencies *f*_*stim*_= 20 Hz as well as *f*_*stim*_ = 22 Hz and varying radiant flux considering only VT events. Figure 3B top and bottom display the success rates of the row and column protocol exceeding those of the array protocol. It is important to notice that the column illumination pattern obtained the highest success rates with the lowest applied radiant flux Φ = (1.7 ± 0.1) mW and therefore the lowest energy of stimulation *Q*= (0.61 ± 0.04) mJ for *f*_*stim*_ = 20 Hz. Conversely, the row illumination pattern delivered better results with increasing radiant flux and stimulation energy in both cases. Both the column and the row protocol reached a 100 % success rate for a certain parameter combination thereby providing a reliable mechanism for arrhythmia termination.

Supplementary Figure 3 displays the success rates for each illumination pattern in dependence on the dominant frequency of the arrhythmia *f*_*arr*_ 1 s before illumination considering all VT events. In this case, the dominant frequency *f*_*arr*_ was determined as the weighted average of the results of the sine fit, the FFT, and the Lorentz fit to the FFT spectrum. Moreover the distribution of *f*_*arr*_ can be found in Supplementary Figure 4. The presented results show that the success rate of the row protocol declined with increasing *f*_*arr*_. Conversely, the column and array protocol showed no such trend. Overall, the array protocol reached the highest success rate only in one case, namely for *f*_*arr*_=21 Hz. In all the other cases, the success rates of either the row or column protocol exceeded that of the array illumination pattern.

Additionally, experimental validation regarding the possibility of self-termination was performed by inducing arrhythmia with the μLED arrays in place, but without applying photostimulation. Given that the arrhythmia was sustained for at least 5 s, it persisted for (30.97 ± 2.50) s for all arrhythmia *N*_*arr*_ = 53 evaluated. Subsequently, the arrhythmia was terminated using global illumination to prevent permanent damage to the heart due to the ongoing chaotic behavior. Thus, it can be concluded that the induced arrhythmia have a self-termination time above (30.97 ± 2.50) s once they have persisted for more than *t*_*term*_=5 s. Since the stimulation protocols take a maximum of 15 s upon completion, the termination of the chaotic cardiac rhythm can clearly be attributed to the stimulation by the μLED arrays.

### 3.3. Effect of Illumination Patterns During Cardiac Arrhythmia

Optical based stimulation does not induce electrical artifacts due to saturation of the amplifier in the ECG recordings, therefore it was possible to analyze time series of the three acquired uni-polar leads before, during, and immediately after all photodefibrillation attempts. Since the dynamic transients of complex arrhythmia could be better characterized with the CWT, the results in this section always use the frequencies *fc* calculated with the method described in section 2.5. Additionally, all types of arrhythmia were included without distinguishing between them during evaluation. In particular, it could be observed that the local dominant frequencies *fc*_*dur*_ notably changed during photodefibrillation independently of the frequency used for photostimulation *f*_*stim*_. Trials in which *fc*_*bef*_ remained constant could not be found, refer to Supplementary Figure 5.

Table 1 shows that during illumination all three tested patterns provoked a change of the mean frequency $\bar{\Delta }fc$ calculated for the three recorded ECG, where Δ*fc* = *fc*_*dur*_−*fc*_*bef*_. This effect is more prominent in the row illumination pattern where 76 % of all arrhythmia displayed a decrease in dominant frequency during photostimulation. Moreover, all three illumination patterns showed to perform better when Δ*fc* was decreased. In other words, when *fc*_*dur*_ decelerates compared to *fc*_*bef*_, the probability of termination success increases, as it is presented in columns 5 and 6 of Table 1. To support this result, the success rates for deceleration and acceleration measured in different uni-polar leads around the heart were extracted and are shown in Figure 3C. The success rates here were organized in four groups, where the first group *dec*._1_ contains the attempts in which deceleration could be observed only in one lead, whereas acceleration was detected in the other two leads. In a similar way, the second group *dec*._2_ contains the tries in which two leads got decelerated, thus one was accelerated. Finally, the last two groups *dec*._3_ and *acc*._3_ show the termination rates when all three uni-polar leads measured deceleration, respectively, acceleration. The most efficient approach for terminating arrhythmia is given when the deceleration of *fc*_*dur*_ is achieved in all three monitored areas of the heart, here the array pattern reached the best efficiency. With exception of the row protocol, the termination rate of the group *acc*._3_ exhibits rather low values.

**Table 1:** Change of average frequency from the CWT during photostimulation where Δ*fc* = *fc*_*dur*_−*fc*_*bef*_.

**Protocol**	***N*_*tot*_**	***N*_Δ*fc*>0_ [%]**	***N*_Δ*fc* <0_ [%]**	***S*_Δ*fc*>0_ [%]**	***S*_Δ*fc* <0_ [%]**	**$\bar{\Delta }fc>0$ [Hz]**	**$\bar{\Delta }fc>0$ [Hz]**
Row	141	34	76	17	83	1.3 ± 1.1	–2.6 ± 1.9
Column	117	37	63	27	72	1.7 ± 1.6	–2.7 ± 2.1
Array	138	47	53	36	64	1.8 ± 1.9	–1.5 ± 1.2

*Shown is the average frequency Δ*fc* resulting from the three uni-polar leads. Columns 3 and 4 display the percentage of arrhythmias that increased or decreased their frequency during photostimulation, respectively. Columns 5 and 6 present the obtained success rates for accelerating and decelerating the heart frequency. In Columns 7 and 8, the amount of change of the average frequency can be seen. It can also be noted that in comparison, the change of frequency $\bar{\Delta }fc$ is higher using row and column pattern than using the array protocol*.

To further analyze this behavior, Figure 3D shows the scatter plots with marginal smoothed histograms of the conducted experiments. The smoothed histograms on the right show that there is an accumulation of successful cases when the frequency of the arrhythmia during photostimulation *fc*_*dur*_ drops below 20 Hz approximately. Directly comparing the three tested patterns, it can be observed that the distribution of terminated arrhythmia in the column protocol is broader than for row and array patterns, demonstrating that the column protocol is more robust against the arrhythmia measured in this work. Furthermore, the right side histograms suggest that the row protocol is more successful when it is able to reduce *fc*_*dur*_ to around 15 Hz, the same claim holds for the array protocol. Considering the upper histograms, it can be observed that column and array protocol perform better for slow arrhythmias.

In order to illustrate the evolution of arrhythmia during photostimulation Figures 3E–G show an example of a termination event for each row, column, and array patterns with corresponding representative uni-polar lead recording, calculated CWT, and the representation of the local change of frequency on the heart *fc*_*dur*_. In Figure 3E, it is shown that the frequency and morphology changes of the measured ECG are clearly noticeable during photodefibrillation. Dashed lines indicate the beginning and end of the light pulses. The frequency and amplitude changes can be seen more pronounced in Figure 3F. These magnitude scalograms show that all three row (left), column (center) and array (right) protocol, modify the initial frequency *fc*_*bef*_ to some extent. Figure 3G emphasizes that uni-polar lead recordings were able to register different cardiac dynamics in the different locations. Moreover, it could also be observed that the change of dominant frequency did not necessarily appear in all regions of the heart during photostimulation. This fact might indicate that the electrical activity of some cardiac tissue was not altered in a significant way. For instance, setting a change of frequency threshold to Δ*fcT* = ± 0.5 Hz for every uni-polar lead, it was found that the employed illumination patterns induced a variation in frequency below Δ*fcT* in only one ECG recording in 19.9 % of the cases for the row protocol, 23.1 % of the total cases in the column protocol, and 29.7 % in the array protocol.

The presented results also point out that the Multi-Lead recording of cardiac activity delivers more spatial information about the dynamics and temporal evolution of an arrhythmia, e.g., during transient events such as during photodefibrillation, than single lead recordings as commonly used.

## 4. Discussion

The motivation for the present study was to demonstrate the great potential of cardiac optogenetics as an investigative tool for elucidating the underlying mechanisms of defibrillation. So far, this question could not be clarified unambiguously, because especially in studies with electrical pulses the ECG is briefly “blind,” i.e., due to the high electrical pulse voltage, the actual cardiac signal is masked. However, with the help of optogenetic channelrhodopsins, here specifically channelrhodopsin-2, no electrical voltage is used for defibrillation, but light pulses, which are suitable in appropriate light intensity to initiate new excitation signals (Bruegmann et al., 2011; Deisseroth, 2011).

In the application of cardiac optogenetics itself, various termination mechanisms are currently being discussed. First and foremost are the physiological processes induced by the applied light intensity, as well as the change of light intensity along the penetration depth into the myocardium (Lubart et al., 2007; Bruegmann et al., 2010; Hussaini et al., 2021). Though, the analysis of the development of the arrhythmia frequency is often limited to the required termination time, so that conclusions can be drawn about the minimum required pulse duration or stimulation duration, but not about the underlying termination process. To try to understand the defibrillation mechanisms, the authors tested three different multi-pulse protocols and analyzed them with respect to arrhythmic behavior during photostimulation. It was shown that the probability of successful arrhythmia termination is strongly dependent on the frequency evolution. With decreased arrhythmia frequency (deceleration), the probability of success is significantly increased compared with increasing frequency (acceleration). Such frequency dependence of defibrillation has also been shown in other pre-clinical or clinical studies with electrical stimulation (Everett et al., 2001; Panfilov et al., 2013). Similarly, the dependence of the probability of success on the pacing frequency used has been demonstrated in other works. For example, Weinberg et al. (2013) tested high-frequency stimulation protocols (50–500 Hz) and identified conduction block as a putative mechanism (Tandri et al., 2011). That is, cardiomyocytes are globally maintained in the refractory phase until the arrhythmia is terminated. Such type of experimental studies could possibly be equated with long-time global photostimulation, such as it can be applied in the array protocol presented here or by Quiñonez Uribe et al. (2018). Nevertheless, such a mechanism is apparently not ostensible for the other two protocols, row and column. Here, the local influence of light-induced depolarization on excitation propagation and thus on the complexity of arrhythmia propagation seems to play a crucial role. These two protocols are a very good example of the use of optogenetic photodefibrillation. The idea hereby is simple, through the targeted local application of light pulses, the arrhythmic excitation pattern is counteracted by a new motion pattern.

As shown in Figure 3A, the different pulse protocols can be used for different arrhythmia types. With regard to automated feedback pacing, this means that different pulse protocols could be applied based on the frequency analysis and arrhythmia classification preceding the termination pacing, thus increasing the termination probability. This was also confirmed for conventional defibrillation by Povoas et al. (2002) by analyzing pre-clinical data for CPR. It is important to mention that, in order to exclude effects in the success rate analysis due to spontaneous self-termination, experiments were performed with the same setup except for the activation of the μLED arrays. The experiments have shown that the probability of self-termination decreases with the duration of the induced arrhythmia (see section 3.2). Based on this, the authors assume that the terminations shown here occurred mainly because of photostimulation. Bruegmann et al. (2016) have shown that both ChR2 expression by itself and pure illumination of wild-type hearts have no effect on termination rates. Taking this into account, the authors decided not to use such a control group in the present work. Although this study provides the proof of principle that different illumination patterns do influence the termination success of an arrhythmia depending on its complexity (mVT, pVT, or VF), the necessity for further testing remains. Based on this, there is plenty of potential for optimization with the aim of establishing specific termination parameters based on arrhythmia characteristics and applying these dynamically during the experiment so that each event can be treated in a customized manner. More precise defibrillation mechanisms cannot yet be represented by the data generated in this study and are therefore object of ongoing research.

In addition, it could be successfully proven that the integration of simultaneous Multi-Lead ECG measurements in combination with frequency and time-frequency analyzes allows a better spatial characterization of arrhythmia compared to conventional one lead ECG, without the need of using optical mapping. Consequently, the design and development of targeted termination pulses with patterned illumination were made possible. It is also important to mention that the use of a triangular constellation of measuring electrodes as shown here gives a better understanding of the arrhythmia distribution, but for the precise spatial classification of the arrhythmia only rather large-scale statements can be made (see Figure 3G). To refine this, further studies in the group are looking at the use of new ECG arrays with higher spatial resolution. Further examples from the literature comply with the intended way to follow (Uzelac and Fenton, 2020).

### 4.1. Limitations and Outlook

The present study describes a possible dependence of the termination success on the development of the arrhythmia frequency during pacing. Though, at the present time, no conclusions can be drawn about the underlying excitation patterns. The complexity of arrhythmia and, consequently, the arrhythmia frequency may change spatially due to meandering spirals. To investigate this, the number of ECG electrodes used is not sufficient. For this, the electrode density needs to be increased in relation to the heart surface. Another method to overcome this challenge would be the usage of optical mapping, however, while it is true that it is widely accepted as the standard technique for the electrical spatial characterization of arrhythmia, it can also produce non-desired electrophysiological changes as shown by Kappadan et al. (2020). Other methods like combined optical mapping with motion tracking (Christoph et al., 2017, 2018) could also be helpful, but the motion compensation can only be done after the experiments during post-processing. Therefore, and with the motivation of opening the possibility to translate the presented results into *in vivo* trials or also bigger animal models, the authors decided to introduce the Multi-Lead ECG measurement as a local monitor of cardiac electrical activity. Further efforts are being invested in the real-time processing of the acquired ECG measurements. While optical mapping data requires much more computational power, ECG measurements can be directly analyzed during trials with lower processing resources such as microcontrollers or digital signal processors, facilitating the near to instantaneous analysis of measured data.

A somehow obvious limiting factor of the present study is the currently difficult translation of the described technique into clinically relevant *in vivo* applications. For this purpose, in addition to the above mentioned optimizations of the electrical measurement methods, anatomically adapted measurement and stimulation arrays are needed, the detailed study of which is the subject of current investigations.

Further limitations arise from the optical characteristics of the μLED used, such as the beam angle. This affects the distribution of the emitted light intensity on the cardiac surface and thus the propagation mode of the initiated excitation. Characterization of the propagation parameters would be highly important for understanding the termination processes and for establishing a feedback system based on them. Therefore, increasing ECG resolution and the study of μLED light distribution patterns in combination with optical visualization as a validation method is part of ongoing research.

With the intention of finding parameters that might predict the termination of an arrhythmia even before the photodefibrillation attempt is finished, the CWT processing method was introduced in this work as an alternative method to the FFT for determining the dominant frequency. It could successfully be shown that the CWT facilitates the characterization of arrhythmia frequency during short events of light stimulation. Further looking for prediction parameters, Yang et al. (2019) investigated whether the amplitude spectrum area (AMSA) and spectral energy could be considered indicative for the success of subsequent attempts at arrhythmia termination. As a result, they found that both parameters significantly increased in cases that lead to successful defibrillation. In contrast, if defibrillation did not succeed, no increase in AMSA or spectral energy could be observed. Likewise, in the present study, a change in the amplitude of the ECG recording of arrhythmia was noticed in some cases. Based on these two approaches (frequency and amplitude analysis) and the presented arrhythmia classification method, the design of a new feedback protocol is being pursued, with the goal of improving the photodefibrillation outcome by evaluating the mentioned parameters in real-time.

## 5. Conclusion

In conclusion, the presented experimental setup and the analyses obtained from it so far are the first step toward the description of arrhythmic development during multi-pulse photostimulation, which contributes to the successful termination due to the better resolution in spatial and temporal information, as well as better classification of the occurring arrhythmia. Further protocol flexibility is given by the fact that the proposed setup is able to individually control single μLEDs rather than only follow rigid illumination patterns, providing a fast theory-to-proof to experimental-implementation time. The authors are convinced that for the elucidation of defibrillation mechanisms, cardiac optogenetics is a well-suited experimental tool. Even so, there are still a few hurdles along the way, such as linking the arrhythmia frequency to the light intensity used and the associated change in amplitude or integrating flexible pacing frequencies.

## Data Availability Statement

The original contributions presented in the study are included in the article/Supplementary Material, further inquiries can be directed to the corresponding authors.

## Ethics Statement

The animal study was reviewed and approved by Lower Saxony State Office for Consumer Protection and Food Safety (LAVES) Dezernat 33-Tierschutzdienst Postfach 3949 26029 Oldenburg.

## Author Contributions

LD-M and JS performed and analyzed the experiments. LD-M and CR designed research and experiments. LD-M, JS, and CR conceptualized, wrote, and edited the manuscript. All authors agree to be accountable for the content of the work.

## Funding

The support was provided by the DZHK e.V., the German Federal Ministry of Education and Research (BMBF, project FKZ 031A147, Go-Bio), the German Research Foundation (DFG, Collaborative Research Centers SFB 1002, Projects B05 and C03), and the Max Planck Society.

## Conflict of Interest

The authors declare that the research was conducted in the absence of any commercial or financial relationships that could be construed as a potential conflict of interest.

## Publisher's Note

All claims expressed in this article are solely those of the authors and do not necessarily represent those of their affiliated organizations, or those of the publisher, the editors and the reviewers. Any product that may be evaluated in this article, or claim that may be made by its manufacturer, is not guaranteed or endorsed by the publisher.
