# Supplementary material for: Patterned Illumination Techniques in Optogenetics: An Insight Into Decelerating Murine Hearts
Source: Front Physiol. 2022 Jan 11;12:750535. doi: 10.3389/fphys.2021.750535 (PMC8787046; doi:10.3389/fphys.2021.750535)
Supplement: Supplementary file 1 [file Data_Sheet_1.pdf]

Supplementary Material : Diaz-Maue et al.

1 SUPPLEMENTARY TABLES AND FIGURES

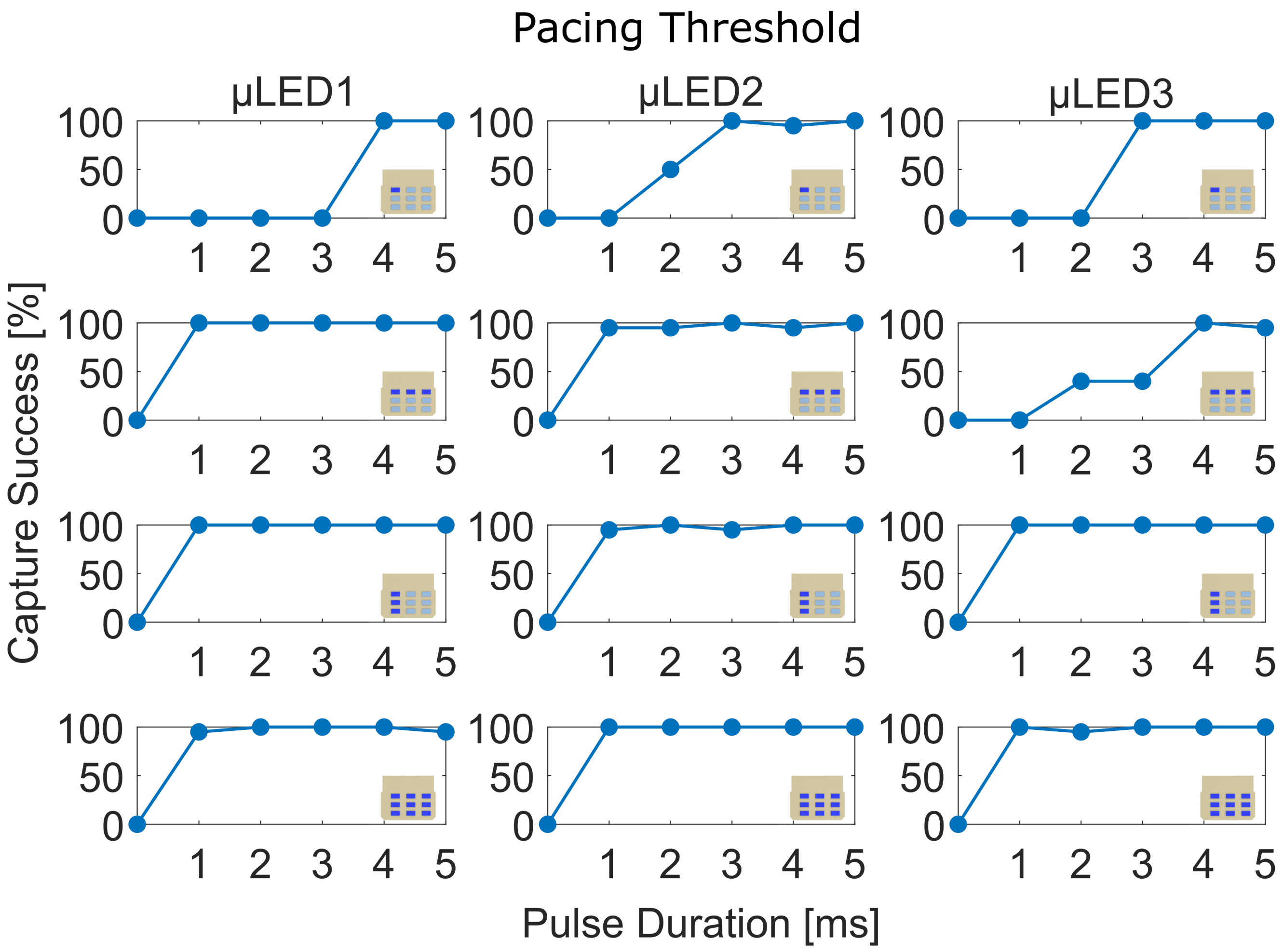

Figure S1. Measured pacing threshold for $N = 1$ murine heart perfused with Low-K+ Tyrode's Solution using the three μLED arrays arranged as shown in Figure 1. Every pacing attempt used the following parameters, radiant flux $\Phi = 1.7\,\mathrm{mW}$, pulse number $k = 20$, pacing frequency $f_{pac} = 8\,\mathrm{Hz}$, pulse width $d_{pac} = 1\,\mathrm{ms}$, $2\,\mathrm{ms}$, $3\,\mathrm{ms}$, $4\,\mathrm{ms}$ and $5\,\mathrm{ms}$ and the number of μLED as shown in the inlets of every plot. The first row of plots shows that illuminating with only one μLED yields to$100\,\%$ capture from $3\,\mathrm{ms}$ on and from $4\,\mathrm{ms}$ on in the case of μLED1 . Furthermore, the last row of plots shows that all the tested d_{pac} achieved $100\,\%$ capture when using nine μLEDs of every array

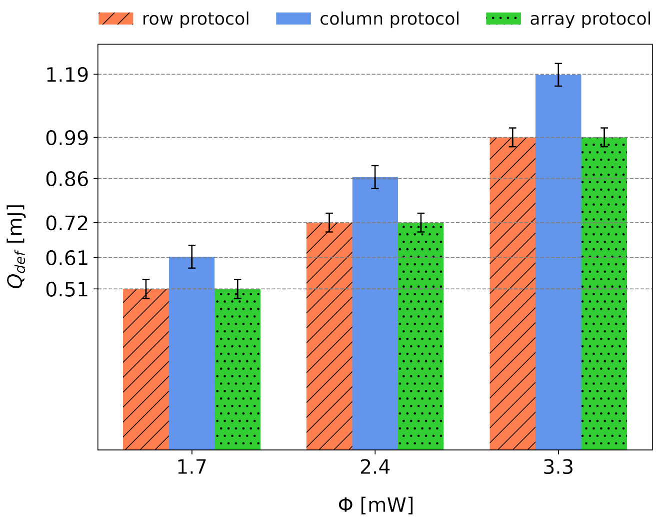

Figure S2. The energies of photostimulation $Q_{def} = k \cdot \Phi \cdot d_{stim}$ for the different parameter combinations used in the present study. k is the number of pulses, d_{stim} =20 ms the pulse duration and Φ the radiant flux applied.

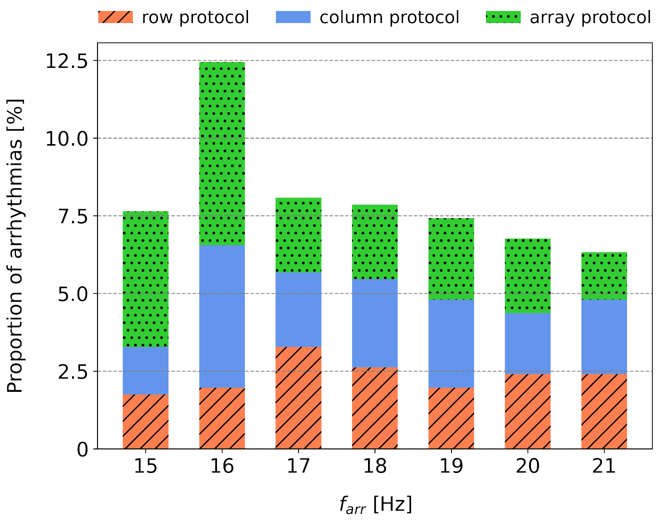

Figure S3. The distribution of the dominant frequencies of the arrhythmia f_{arr} determined as described in Section 2.4. f_{arr} was evaluated 1 s before photostimulation.

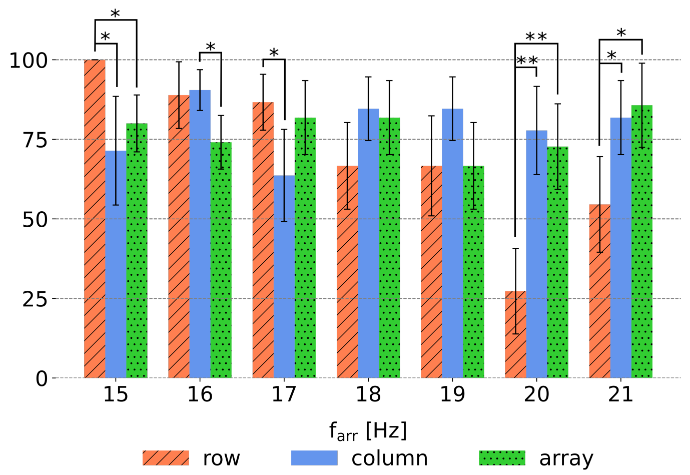

Figure S4. Distribution of success rates of the three different pacing protocols shown in dependency to the calculated f_{arr}. Error bars shown as Standard Error of Mean (SEM). Statistical significance displayed as $*p$ <0.1, $**p$ <0.05 and $***p$ <0.01.

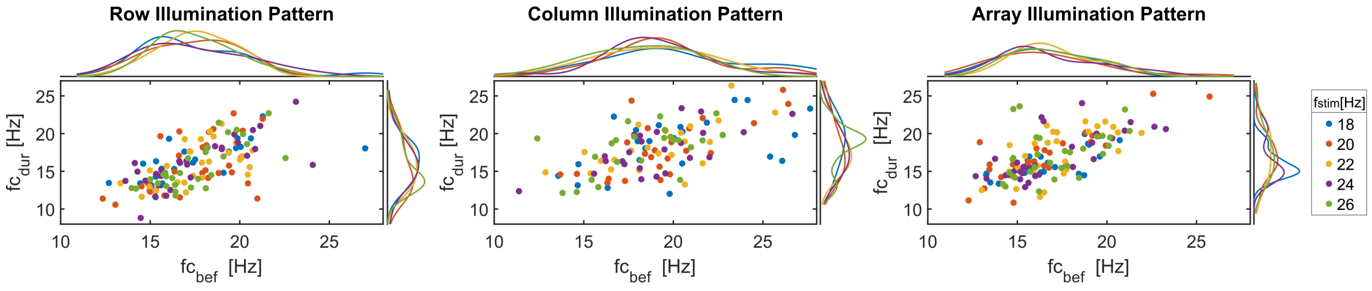

Figure S5. Distribution of the calculated arrhythmia frequencies during photodefibrillation fc_{dur} against the arrhythmia frequency before stimulation fc_{bef}. Both frequencies were calculated as described in Section 2.5. It can be seen that all three illumination patterns produce a change of frequency fc_{dur} independently of the applied f_{stim}.
